# Supplementary material for: Direct band-gap crossover in epitaxial monolayer boron nitride
Source: Nat Commun. 2019 Jun 14;10:2639. doi: 10.1038/s41467-019-10610-5 (PMC6572751; doi:10.1038/s41467-019-10610-5)
Supplement: Supplementary file 1 — Supplementary Information [file 41467_2019_10610_MOESM1_ESM.pdf]

Supplementary Information for

Direct band-gap crossover in epitaxial monolayer boron nitride

by C. Elias *et al.*

Supplementary Note 1. MOLECULAR BEAM EPITAXY

1.1 Growth

We recently demonstrated that with molecular beam epitaxy (MBE) we can control the hBN layer coverage by adjusting the epitaxial growth temperature (T_g) in the range from 1390 to 1690 °C [S1,S2]. We thereby produced partial coverage of the graphite substrate by monolayer hBN. At the highest growth temperatures only a small density of hBN islands was obtained. The coverage gradually increased to a complete boron nitride monolayer (mBN) by decreasing the growth temperature.

Supplementary Figure 1 presents an alternative way to control the hBN coverage by increasing the boron flux during the MBE growth. The hBN layers were grown at the same temperature, T_g ~ 1390 °C, on highly oriented (sometime named highly ordered) pyrolitic graphite (HOPG) substrates with a fixed active nitrogen flux and a growth time of 3 hours. An increase in the temperature of the boron source (T_B) results in an increased boron flux and therefore an increase in the hBN growth rate. We quote the temperature of the boron cell, but not the boron beam equivalent pressures (BEP) because we were not able to achieve reliable measurements of the boron flux with a beam-monitoring ion gauge [S3]. The boron flux depends exponentially on the boron cell temperature (in degrees K). Therefore, we expect a very gradual increase in the hBN thickness for the first three layers [Supplementary Fig.1(a-c)] with an abrupt increase for the last two samples in Supplementary Fig.1(d&e).

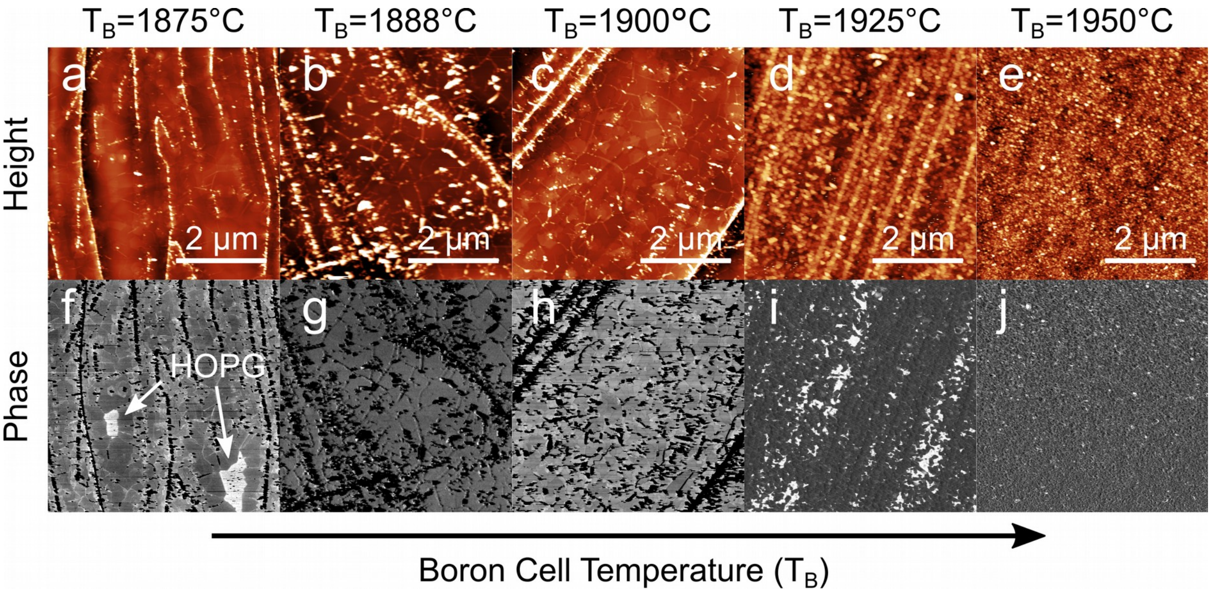

Supplementary Figure 1. Tapping-mode ambient AFM images of MBE-grown hBN on HOPG substrates for increasing boron cell temperatures (T_B) from left to right (T_g = 1390 °C and growth time of 3 hours). a-e) Topographic height-channel images for increasing T_B show the increase in surface coverage from sub-monolayer (a) to complete monolayer coverage (b) and a rough surface for the thickest hBN layer (e). f-j) Phase-channel data for the images shown in a-e. The white arrows in image f indicate two exposed regions of HOPG that remain uncovered by hBN growth (bright contrast regions).

We used variable angle spectroscopic ellipsometry (VASE) to study the thickness of the hBN layers. The VASE was carried out using a M2000-DI instrument made by J.A. Woollam, Inc [S3]. The results were obtained over a wavelength range from 1690 to 192 nm using focussing probes which result in an elliptical spot with a minor axis of 200 μm; the major axis of the ellipse depends on the angles of incidence, which were 55°, 60°, and 65°. Analysis was carried out using CompleteEase version 5.19. The mosaic spread of the HOPG substrate means that we need to allow for an angular offset in the ellipsometric models. The average thicknesses of the three samples grown at boron cell temperatures between 1875 and 1900 °C [Supplementary Fig.1(a-c)] were less than 1 nm. However, we are not able to determine the exact number of hBN monolayers in these three layers because the level of uncertainty for VASE over this thickness range is about 0.5 nm. The average thicknesses of the hBN layers grown at the higher boron cell temperatures rapidly increased to ~3 nm (T_B=1925 °C) and ~32 nm (T_B=1950 °C), Supplementary Fig.1(d) and Supplementary Fig.1(e) respectively.

To characterise the surface structure of MBE-grown hBN, we performed tapping mode (AC-mode) atomic force microscopy (AFM) measurements of the as-grown hBN on HOPG.

For the lowest T_B shown in Supplementary Fig.1a (1875 °C) the HOPG surface is almost completely covered by monolayer boron nitride (mBN) growth with the exception of a few small regions of exposed HOPG [see phase-channel image in Supplementary Fig.1(f)] and some hBN aggregates at HOPG step edges [brightest contrast in Supplementary Fig.1(a)]. For increasing T_B [Supplementary Fig.1(b)] the surface becomes completely covered by mBN with no identifiable voids in the mBN layer. With a further increase of the boron flux we observed an increase in the nucleation of regions of multi-layer hBN, as shown in Supplementary Fig.1(c). This is accompanied by an increase in nucleation of 3D hBN deposits from terrace step-edges which cover the surface for T_B=1925 °C [Supplementary Fig.1(d)]. We observed a rough surface for the thickest hBN film [Supplementary Fig.1(e), T_B=1950 °C].

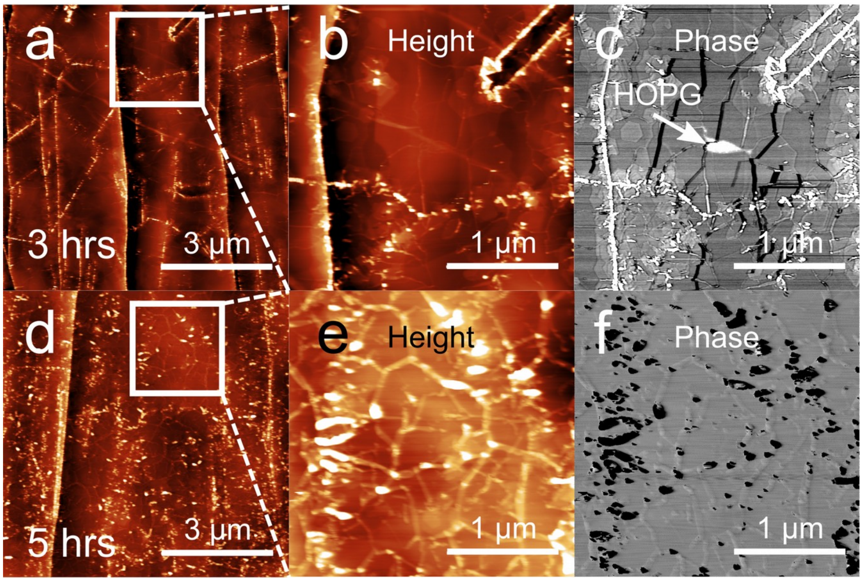

Supplementary Figure 2. AFM images of MBE-grown hBN on HOPG for different growth times (T_B = 1875 °C, T_g = 1390 °C). a) hBN growth on HOPG after 3 hours, the brighter (i.e. topographically higher) regions are due to 3D BN aggregates nucleating at HOPG step edges. b) Zoom of the region indicated by the white box in a. c) Phase-channel data for image b. The white arrow indicates the position of an exposed region of HOPG uncovered by mBN growth. d) Topography of hBN on HOPG after 5 hours of growth showing increased aggregation of hBN deposits. e) Zoom of the region indicated by the white box in d. f) Phase-channel data for image e, the black dots correspond to the 3D aggregates of hBN and there are no uncovered HOPG regions on the surface.

Another way to control reproducibly the hBN coverage is to change the MBE growth time. Supplementary Fig.2 presents AFM images of the hBN islands grown at 1390 °C on a HOPG surface for fixed boron and nitrogen fluxes as a function of the deposition time. For

the hBN layer grown during 3 hours [Supplementary Fig.2(a-c)] we observe coalescence of the hBN islands and the formation of one monolayer boron nitride. There are still small areas of the open uncoated HOPG surface, as indicated in Supplementary Fig.2(c), so the hBN coverage is still slightly below 1 monolayer. One can also observe the formation of bi- and tri-layer hBN along the HOPG step edges [Supplementary Fig.2(c)]. With further increase of the growth time up to 5 h, it is possible to achieve complete coalescence and complete coverage of mBN which is shown in Supplementary Fig.2(d-f). However, at this stage we also observe the formation of bi-, tri-hBN layers and 3D material growth on the surface, partially due to the twist between HOPG plates.

1.2 Dielectric permittivity of graphite

In the context of DUV optoelectronics, graphite appears to be very suitable as an optical substrate for BN. This is a major asset since it is an almost lattice-matched growth template for mBN. The dispersion of the real (ϵ_1) and imaginary (ϵ_2) parts of the dielectric permittivity of graphite measured in Ref.[34] of the main text are plotted in Supplementary Fig.3. Of particular interest is the decrease of ϵ_2 above 4.5 eV, corresponding to a reduction in the optical absorption of graphite in the DUV.

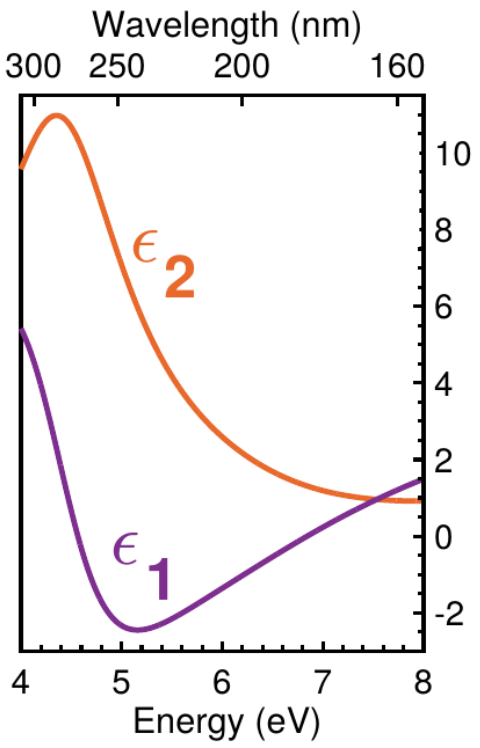

Supplementary Figure 3. Real (ϵ_1) and imaginary (ϵ_2) parts of the dielectric permittivity of graphite, from Ref. [34] of the main text. Note the low-loss property of graphite in the deep ultraviolet, with the strong decrease of ϵ_2 above 4.5 eV (below 275 nm).

Supplementary Note 2. ORIGIN OF THE SECONDARY MINIMUM AT 6 eV IN THE REFLECTANCE SPECTRUM

The existence of a secondary minimum at 6 eV in the reflectance spectrum of mBN on graphite [blue line, Fig.2(a) in the main text] was highlighted in the main text, and tentatively interpreted as resulting from an extrinsic effect, namely a defect-induced brightening of the triplet dark exciton, or as trion absorption.

The extrinsic origin of the 6 eV-peak was deduced from the sample-dependent visibility of this secondary minimum in reflectance spectroscopy. On the one hand, the absolute minimum at around 6.11 eV is observed reproducibly in different mBN samples [Supplementary Fig.4], with the same position in energy and the same absolute level of reflectance. On the other hand, the visibility of the secondary minimum at 5.98 eV depends on the growth conditions, as shown in Supplementary Fig.4. While Supplementary Fig.4(a) corresponds to Fig.2(a) in the main text, Supplementary Fig.4(b&c) display the reflectance measurements for epilayers mostly composed of mBN, but with slightly different surface morphology (see section A). The 5.98 eV-peak is still resolved in Supplementary Fig.4(b) but with a lower contrast and broader lines. In the case of Supplementary Fig.4(c), 5.98 eV-peak is hardly observable, appearing as a low-energy shoulder of the main resonance around 6.11 eV.

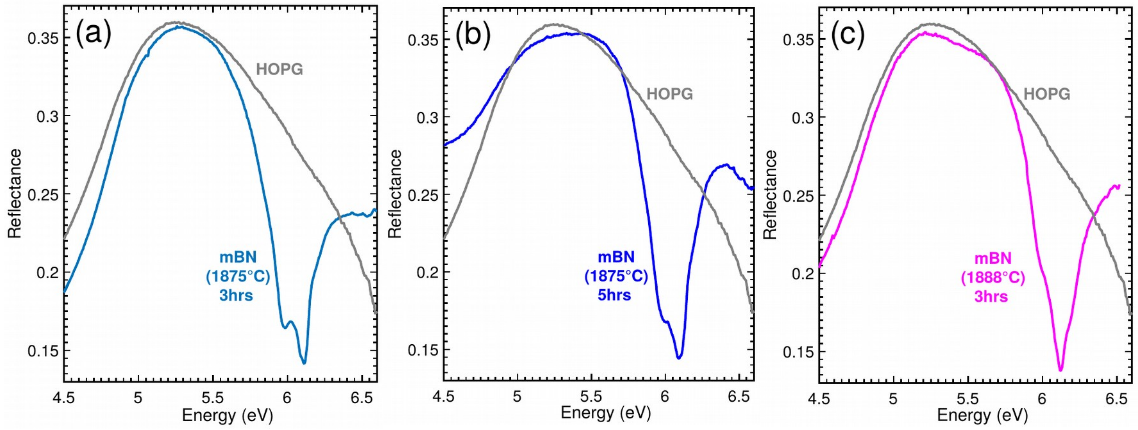

Supplementary Figure 4. DUV reflectance spectrum for several mBN samples measured at 10 K, obtained for different MBE boron cell temperatures (T_B) and growth times : (a) T_B=1875°C, 3 hours, (b) T_B=1875°C, 5 hours, (c) T_B=1888°C, 3 hours. The solid grey line is the reflectance spectrum of the bare HOPG substrate for reference.

Different possible scenarios can account for the existence of a secondary minimum at 6 eV.

2.1 Defect-induced brightening of the triplet dark exciton

The 130 meV-splitting between the two reflectance peaks in Supplementary Fig.4 suggests the involvement of a triplet dark exciton, an observation that was reported in carbon nanotubes as a consequence of a defect-induced brightening [S4,S5]. In addition to the valley degeneracy discussed in the main text, the inclusion of the spin degree of freedom leads to 16 excitonic states [S6] : 12 correspond to a spin triplet, and the other 4 to a spin singlet. The latter are the four states discussed in the main text in the context of intra-valley and inter-valley excitons.

In a perfect crystal, the spin triplet states are optically inactive, so their observation requires some symmetry-breaking mechanism. In carbon nanotubes, it was demonstrated that the presence of defects or distortions efficiently brightens these forbidden states via disorder-induced mixing of singlet and triplet states [S4,S5]. In analogy to the case of carbon nanotubes, we interpret the existence of a secondary minimum at 5.98 eV in the reflectance measurements of mBN epilayers as resulting from a defect-induced brightening of the triplet dark exciton. We note that the 130 meV-splitting in Supplementary Fig.4 lies in the same energy range as for carbon nanotubes [S4,S5]. The reduced visibility of the secondary minimum at 5.98 eV [Supplementary Fig.4] is attributed to a line-broadening due to the progressive increase in defect density in the hBN epilayers beyond one monolayer, together with the formation of 3D material growth on the surface (see section A).

The observation of the brightened triplet dark exciton in reflectance measurements further raises the question of its counterpart in photoluminescence (PL) experiments. Whereas the oscillator strength of a transition is the only relevant parameter for reflectance spectroscopy, carrier relaxation times play a key role in the intensity of the PL signal. In particular, for the occurrence of radiative recombination of a triplet exciton, the transition rate from the singlet to the triplet exciton states, usually called the intersystem crossing rate, should compete with the radiative lifetime of the singlet. In carbon nanotubes, the intersystem crossing time was shown to depend on the defect density, with values ranging from µs to ns [S5]. Although it is difficult to compare with boron nitride, these numbers give an indication of the typical timescale of intersystem crossing in hexagonal crystals. Since phonon-assisted recombination occurs on a sub-ns timescale in indirect-band-gap bulk hBN [S7-S11], one can expect a faster sub-ns recombination in mBN. In fact, radiative recombination of the singlet exciton is likely to be more efficient than intersystem crossing in

mBN, thus leading to a weak PL signal. In Fig.3(a) of the main text, the weak emission line at around 6 eV may be the signature for the low radiative recombination of the brightened triplet exciton.

2.2 Trion absorption

Although the first scenario provides a consistent picture of Supplementary Fig.4, one cannot exclude alternative interpretations, in particular trion absorption being the origin of the secondary minimum at 6 eV in the reflectance spectrum.

A trion is a three-particle bound state consisting of two electrons and a hole, or one electron and two holes. The contribution of trions in the optical response of semiconductors is well documented in the literature. Assuming that the ratio of trion to exciton binding energies in mBN is similar to the value of 10 % found in monolayer transition metal dichalcogenides [Mak *et al.* Nat. Mater. **12**, 207 (2012)], one could also tentatively interpret the redshifted peak in reflectance in Supplementary Fig.4 as the trion absorption. By varying the sample growth conditions, the different intensities of the trion peak in Supplementary Fig.4 may be due to different levels of growth-induced residual doping, which are known to play a key role in transition metal dichalcogenides [Mak *et al.* Nat. Mater. **12**, 207 (2012)] and also in carbon nanotubes [Matsunaga *et al.* Phys. Rev. Lett. **106**, 037404 (2011)].

Supplementary Note 3. THICKNESS-DEPENDENT PHOTOLUMINESCENCE

The demonstration of the direct-gap in monolayers of transition metal dichacolgenides [S12] was performed using the top-down approach of exfoliating few-layer crystals. After characterization of the number of layers for each exfoliated flake, PL measurements gave evidence for an emission much brighter in monolayers than in bi-, tri- and multi-layers, from which the indirect-to-direct transition of the band-gap was identified.

Here, we employ the bottom-up strategy of molecular beam epitaxy to synthesise wafer-scale monolayers of hBN. As explained in Section A, this growth method allows the reproducible fabrication of high-quality monolayers of boron nitride, but not yet of bi-, tri-hBN layers.

At low growth times or low boron fluxes under optimised high-temperature MBE growth conditions, we can achieve a high density of hexagonal mBN islands nucleated on HOPG [S3]. The nucleation points are primarily along the step-edges of the HOPG; however,

there is also a high density of mBN islands on the HOPG plates. By increasing the amount of BN deposited (either by raising the boron source temperature, T_B, or the growth time), it is possible to achieve coalescence of these mBN islands. Initially, we obtain larger mBN islands, with small areas of the uncoated HOPG substrate, corresponding to a mBN coverage somewhat below 1 monolayer. With a further increase in the amount of BN deposited, we can achieve complete coalescence and complete coverage of mBN, together with the start of the formation of bi-, tri-layers and 3D material growth on the mBN surface. With a further increase in the amount of deposited BN, the bi- and tri-hBN layers start to coalesce with the formation of multilayer hBN. With a further increase in time or boron flux, we can grow several tens of nanometre-thick hBN, but with a relatively rough surface. In this MBE process we can reproducibly monitor the formation of mBN with coverage levels below or around 1 monolayer by using *ex-situ* AFM and VASE (section A). However, at present we are not able to achieve the reproducible production of uniform bi-, tri-hBN layers, partly because we have no *in-situ* or *ex-situ* non-destructive characterisation technique to control the hBN thicknesses on the scale of a few monolayers.

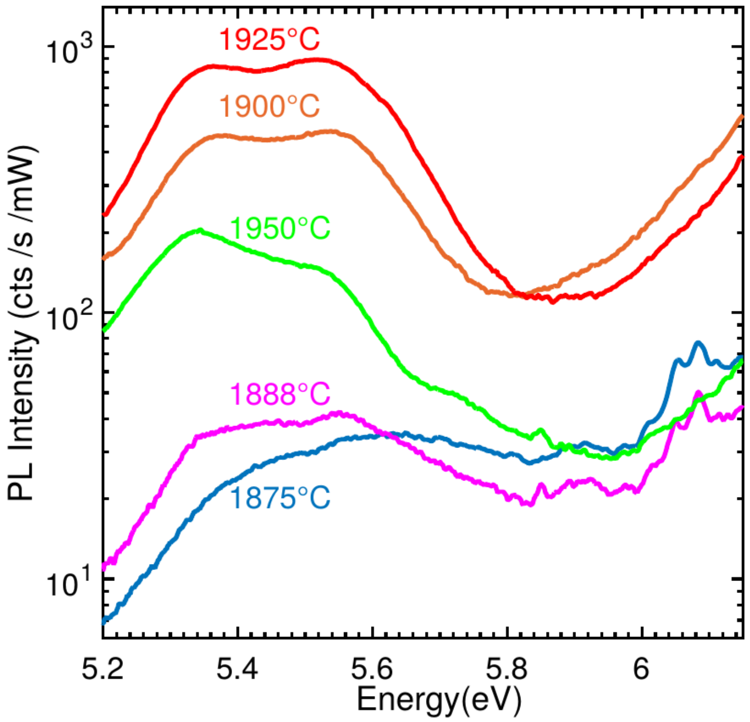

Supplementary Figure 5. PL spectroscopy of hBN epilayers at 10 K, grown with five different MBE boron source temperatures T_B: 1875, 1888, 1900, 1925 and 1950 °C. The spectra are not vertically shifted, and they correspond to the absolute PL signal intensity.

Supplementary Fig.5 displays PL measurements of hBN epilayers grown using five different boron source temperatures T_B, a higher T_B resulted in the larger amount of deposited hBN, see Supplementary Fig.1 in Section A and discussion above.

The PL doublet at 6.05 and 6.08 eV is only detected in the samples, in which the MBE boron source temperature T_B leads to the growth of mBN.

We demonstrated using AFM and VASE studies in Section A that the boron nitride coverage in the sample grown with the boron source temperature of 1875 °C is slightly less than unity [Supplementary Fig.1(a&f)]. We also confirmed with AFM and VASE that the BN coverage for the layer grown with T_B=1888 °C is slightly above one monolayer [Supplementary Fig.1(b&g)]. In both these mBN samples we observe the PL doublet at 6.05 and 6.08 eV associated with the direct band gap of boron nitride [Supplementary Fig.5].

Supplementary Fig.5 shows that for the hBN sample grown with T_B=1900 °C we observed no strong PL lines at energies of around 6.1 eV. AFM data for this sample demonstrates an increase in the formation of regions of multi-layer hBN, as shown in Supplementary Fig.1(c&h). At the same time, according to VASE measurements, the average thickness of this layer is below 1 nm, with level of uncertainty for VASE of ~0.5 nm. Therefore, the average thickness of this layer is about 2 to 3 boron nitride monolayers. We are not able to be more precise on the number of monolayers, because we currently lack *in-situ* or *ex-situ* non-destructive characterisation to determine the hBN thicknesses on the scale of a few monolayers. From the above PL data we conclude that this hBN sample (T_B=1900 °C) already has an indirect band gap.

Supplementary Fig.5 demonstrates that there are no PL peaks at ~6.1 eV and therefore no direct band gap transitions in the hBN sample grown with T_B=1925 °C. We have shown in Section A that this sample has an average thickness of ~3 nm, which corresponds to a thickness of about 10 boron nitride monolayers. Similarly, the thickest hBN layer grown at T_B=1950 °C has an average thickness of ~32 nm. For these two samples, there are no PL lines in the energy range around 6.1 eV. Moreover, Supplementary Fig.5 shows that the intensity of

the defect-related emission band around 5.4 eV increases with the boron source temperature T_B. For the sample grown with T_B=1950 °C, we observe a decrease of the PL intensity, in qualitative agreement with our previous study [S13] reporting a decrease of the PL intensity with increase of the hBN film thickness.

This data demonstrates that the PL doublet at 6.05 and 6.08 eV, associated with a direct band gap, can be observed only in monolayer-thick boron nitride (mBN) samples. The direct band gap transition disappears for hBN layers with thickness of 2 to 3 monolayers and was not detected in the even thicker hBN layers. At present we conclude that such direct-indirect band gap transition occurs at the hBN thicknesses of about 2 to 3 monolayers.

Supplementary Note 4. RESONANT EXCITATION OF PHONON MODES

Because of the giant exciton binding energy in mBN, optical pumping requires the use of resonant excitation of phonon modes. Optical pumping is achieved by a detuning between excitation and detection corresponding to an energy of one or several phonons. This selective optical pumping is the key for detecting the PL signal in mBN.

As discussed in the main text, there is a distortion of the emission spectrum on varying the excitation energy E_{ex}, resulting from the superposition of PL with resonant Raman scattering (RRS), the latter occurring 312 meV below the excitation energy. To better resolve the contribution of RRS, Supplementary Fig.6 plots the PL spectra for E_{ex}=6.4, 6.39 and 6.375 eV in separate panels, and compares each with the PL spectrum for E_{ex}=6.345 eV, displayed as a dotted line.

For E_{ex}=6.345 eV, mBN is excited under off-resonance conditions, resulting in a weak signal intensity, approximately one order of magnitude lower, but dominated by the contribution of PL. We conclude that for E_{ex}=6.345 eV, the ~1.33 intensity ratio between the 6.05 and 6.08 eV lines reflects the genuine aspect ratio of PL.

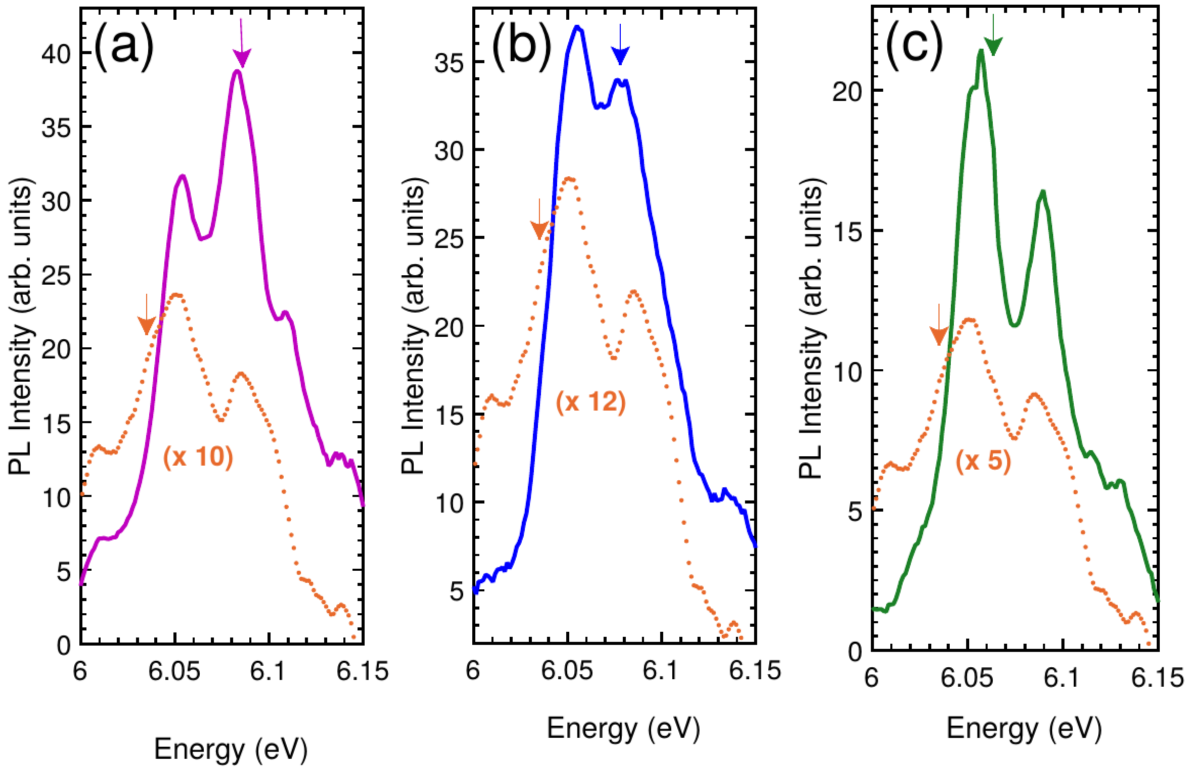

Supplementary Figure 6. PL emission of mBN (after subtraction of stray laser light) for different values of the excitation energy E_{ex} : (a) 6.4, (b) 6.39, (c) 6.375 eV. The dotted line is the emission spectrum for E_{ex}= 6.345 eV, multiplied by a scaling factor indicated in brackets. The vertical arrows indicate the Raman-shifted energy E_{ex} - 2Δ, with Δ=156 meV, corresponding to the energy of the LA(M) phonon in mBN.

For E_{ex}=6.375 eV [Supplementary Fig.6(c)], the 1.3 intensity ratio is comparable to the one recorded for E_{ex}=6.345 eV, from which we conclude that the RRS contribution is still low. This is in agreement with our interpretation of the emission doublet, where the 6.05 eV line corresponds to phonon-assisted recombination of the momentum-dark exciton in mBN, implying that there is no real exciton state at the energy of 6.05 eV, and thus no significant RRS for this excitation energy.

In contrast, for E_{ex}=6.4 eV [Supplementary Fig.6(a)], we find RRS with the direct exciton at 6.08 eV so that RRS makes an important contribution to the measured spectrum. In that case, the intensity ratio between the 6.049 and 6.083 eV lines reverses from 1.33 to 0.8.

For the intermediate case of E_{ex}=6.39 eV, there is a slight detuning from the RRS condition with the direct exciton : the maximum of the emission spectrum no longer corresponds to the energy of the direct exciton at 6.08 eV, but rather to the red-shifted RRS signal.

Finally, we highlight an important difference between our measurements in mBN and the resonant excitation experiments reported for $MoSe_2$ in Ref.[S14], namely the RRS linewidth compared to that of the PL. In Ref.[S14], excitation spectroscopy was performed with a tunable cw laser, so that the RRS linewidth was much smaller than that of the excitonic PL. Hence, one can easily discriminate between RRS and PL from their respective linewidth in Ref.[S14].

In our experiment, the mBN samples are excited with femtosecond pulses from the fourth-harmonic of a cw mode-locked Ti:Sa oscillator. Rayleigh scattering of the excitation source gives a broad line, with a full width at half maximum (FWHM) of ~9 meV. This value is roughly three times smaller than the FWHM of the doublet lines (see Fig.3(d) in the main text, and section E below). However, since the RRS signal arises from the emission of 2LA(M) phonons, we are dealing with second-order Raman scattering, for which we expect a FWHM twice that of the Rayleigh scattering FWHM : the intrinsic Raman broadening due to the phonon lifetime is negligible under our excitation conditions with femtosecond pulses. The result is an RRS line-width of ~18 meV, comparable to that of the PL lines in the 6.05 and 6.08 eV doublet, as observed in Supplementary Fig.6.

Supplementary Note 5. TEMPERATURE-DEPENDENT PHOTOLUMINESCENCE

We performed temperature-dependent measurements in order to study the impact of the giant exciton binding energy and atomically-thin structure on the opto-electronic properties of mBN.

5.1 Quantitative analysis of the experimental data

Supplementary Fig.7 displays the measurements (open circles) of the emission spectrum from 8 to 300 K, in the energy range of interest, corresponding to the doublet at 6.05 and 6.08 eV. All spectra in Supplementary Fig.7 have been corrected for the background arising from laser light scattering.

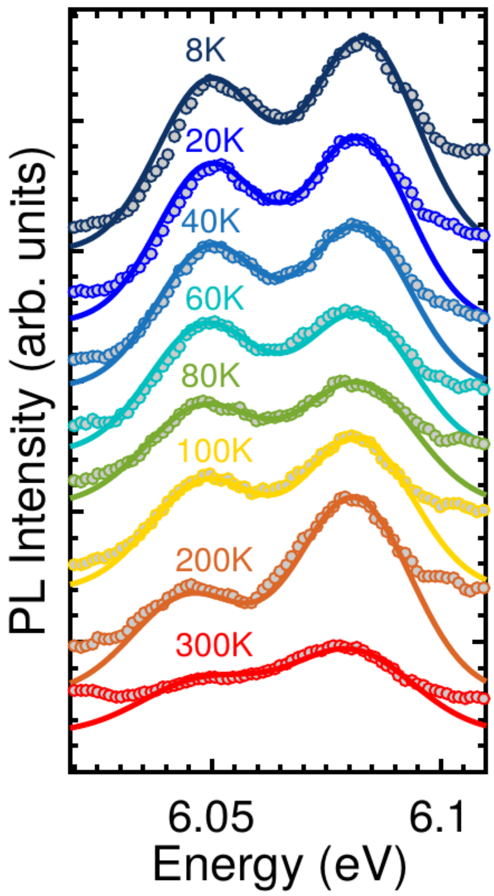

Supplementary Figure 7. PL spectra in mBN as a function of temperature: data (open circles) and fit (solid lines). All spectra have been corrected for the background arising from laser light scattering, and they are vertically shifted for clarity.

To quantitatively analyse our temperature-dependent measurements, we fitted the emission doublet using the sum of two Gaussian functions with the same linewidth, amplitudes A_1 and A_2, and central energies E_1 and E_2 , with E_1~6.049 eV and E_2~6.083 eV. These fitting parameters are adjusted to reproduce our experimental data (fits in solid lines in Supplementary Fig.7), focusing on the central part of the spectrum and avoiding the spurious contributions on the low and high energy sides.

Panel (c) in Fig.3 of the main text displays the temperature variations of the amplitude A_1 normalized to its value at 8 K ; panel (d) shows the temperature variations of the FWHM (Γ) of the Gaussian functions.

5.2 Phonon-assisted broadening

The solid line in Fig.3(d) in the main text is a fit of the thermally-induced broadening according to the expression:

$$\Gamma = \sqrt{\Gamma_0^2 + S_A E_A \coth\left(\frac{E_A}{2k_B T}\right)}$$

with Γ_0 = 32 meV, S_A = 8 meV and E_A = 4 meV. Γ_0 is the broadening in the limit of zero temperature, and the second term is due to quasi-elastic scattering by acoustic phonons of mean energy E_A ; k_B the Boltzmann constant and S_A the coupling strength to acoustic phonons. The square root in the equation above arises from the use of Gaussian functions, the convolution of two Gaussian functions of width Δ_1 and Δ_2 having a width $\sqrt{\Delta_1^2 + \Delta_2^2}$.

The second term is formally identical to the expression used in our previous study on the thermal broadening in bulk hBN [S15]. In Ref.[S15], we demonstrated that bulk hBN displays the two required signatures for a thermal broadening in the strong coupling regime, namely a Gaussian line profile and a square-root temperature dependence of the excitonic linewidth. In line with Ref.[S15], we also use a Gaussian line profile and a square-root temperature dependence of the acoustic phonon broadening to analyse our data.

We note three important differences between mBN and bulk hBN. First, there is no additional term due to broadening by optical phonons, which correspond in bulk hBN to the interlayer breathing mode [S15]. This broadening was dominant above 50 K in bulk hBN, but it is absent in mBN because of the atomically-thin structure of mBN, and the subsequent disappearance of the interlayer breathing mode. Second, there is an additional term (Γ_0) accounting for the linewidth in the limit of zero-temperature. Such a term was absent in bulk hBN, where the linewidth was controlled only by phonon broadening. In the case of mBN, Γ_0 originates from inhomogeneous broadening (in addition to the finite radiative broadening). The presence of this term, and the fact that it dominates over the thermal broadening, strongly reduces the visibility of the square root temperature-dependence of the phonon broadening observed in bulk hBN [S15]. Third, the broadening due to acoustic phonons is slightly higher in mBN than in bulk hBN. Taking for mBN the mean energy E_A of 4 meV found in bulk hBN, we obtain fair agreement with our experimental data [solid line in Fig.3(d) in the main text] with a value of 8 meV for S_A, compare to 4.5 meV for bulk hBN [S15]. This increase is likely to arise from the reduced excitonic Bohr radius of mBN.

* guillaume.cassabois@umontpellier.fr

Supplementary references

[S1] Cho, Y. J. *et al.*. Hexagonal boron nitride tunnel barriers grown on graphite by high temperature molecular beam epitaxy. *Sci. Rep.* **6**, 34474 (2016).

[S2] Vuong, T. Q. P. *et al.* Deep ultraviolet emission in hexagonal boron nitride grown by high-temperature molecular beam epitaxy. *2D Mater.* **4**, 021023 (2017).

[S3] Cheng, T. S. *et al.* High-temperature molecular beam epitaxy of hexagonal boron nitride layers. *J. Vac. Sci. Technol. B* **36**, 02D103 (2018).

[S4] Harutyunyan, H. *et al.* Defect-Induced Photoluminescence from dark excitonic states in individual single-walled carbon nanotubes. *Nano Lett.* **9**, 2010-2014 (2009).

[S5] Nagatsu, K., Chiashi, S., Konabe, S. & Homma, Y. Brightening of triplet dark excitons by atomic hydrogen adsorption in single-walled carbon nanotubes observed by photoluminescence spectroscopy. *Phys. Rev. Lett.* **105**, 157403 (2010).

[S6] Ando, T. & Uryu, S. Theory of excitons in carbon nanotubes. *Phys. Status Solidi (c)* **6**, 173-180 (2009).

[S7] Watanabe, K., Taniguchi, T., Kuroda, T. & Tsuda, O. Time-resolved photoluminescence in band-edge region of hexagonal boron nitride single crystals. *Diam. Relat. Mater.* **17**, 830 (2008).

[S8] Watanabe, K. *et al.* Hexagonal boron nitride as a new ultraviolet luminescent material and its application-Fluorescence properties of hBN single-crystal powder. *Diam. Relat. Mater.* **20**, 849 (2011).

[S9] Cao, X. K., Clubine, B., Edgar, J. H., Lin, J. Y. & Jiang, H. X. Two-dimensional excitons in three-dimensional hexagonal boron nitride. *Appl. Phys. Lett.* **103**, 191106 (2013).

[S10] Cassabois, G., Valvin, P. & Gil, B. Intervalley scattering in hexagonal boron nitride. *Phys. Rev. B* **93**, 035207 (2016).

[S11] Chichibu, S. F., Ishikawa, Y., Kominami, H. & Hara, K. Nearly temperature-independent ultraviolet light emission intensity of indirect excitons in hexagonal BN microcrystals. *J. Appl. Phys.* **123**, 065104 (2018).

[S12] Wang, G. *et al.* Colloquium: Excitons in atomically thin transition metal dichalcogenides. *Rev. Mod. Phys.* **90**, 021001 (2018).

[S13] Vuong, T. Q. P. *et al.* Deep ultraviolet emission in hexagonal boron nitride grown by high-temperature molecular beam epitaxy. *2D Mater.* **4**, 021023 (2017).

[S14] Chow, C. M. *et al.* Phonon-assisted oscillatory exciton dynamics in monolayer $MoSe_2$. *npj 2D Materials and Applications* **1**, 33 (2017).

[S15] Vuong, T. Q. P. *et al.* Exciton-phonon interaction in the strong coupling regime in hexagonal boron nitride. *Phys. Rev. B* **95**, 201202 (2017).
